# Quantitative pharmacology in antibody-drug conjugate development: armed antibodies or targeted small molecules?

**DOI:** 10.18632/oncoscience.435

**Published:** 2018-06-28

**Authors:** Ian Nessler, Eshita Khera, Greg M. Thurber

**Affiliations:** Department of Chemical Engineering, Department of Biomedical Engineering, University of Michigan, Ann Arbor, MI 48109, USA

**Keywords:** antibody-drug conjugate, mechanism of action, immune cell recruitment, payload delivery, receptor signaling

**Figure 1:**
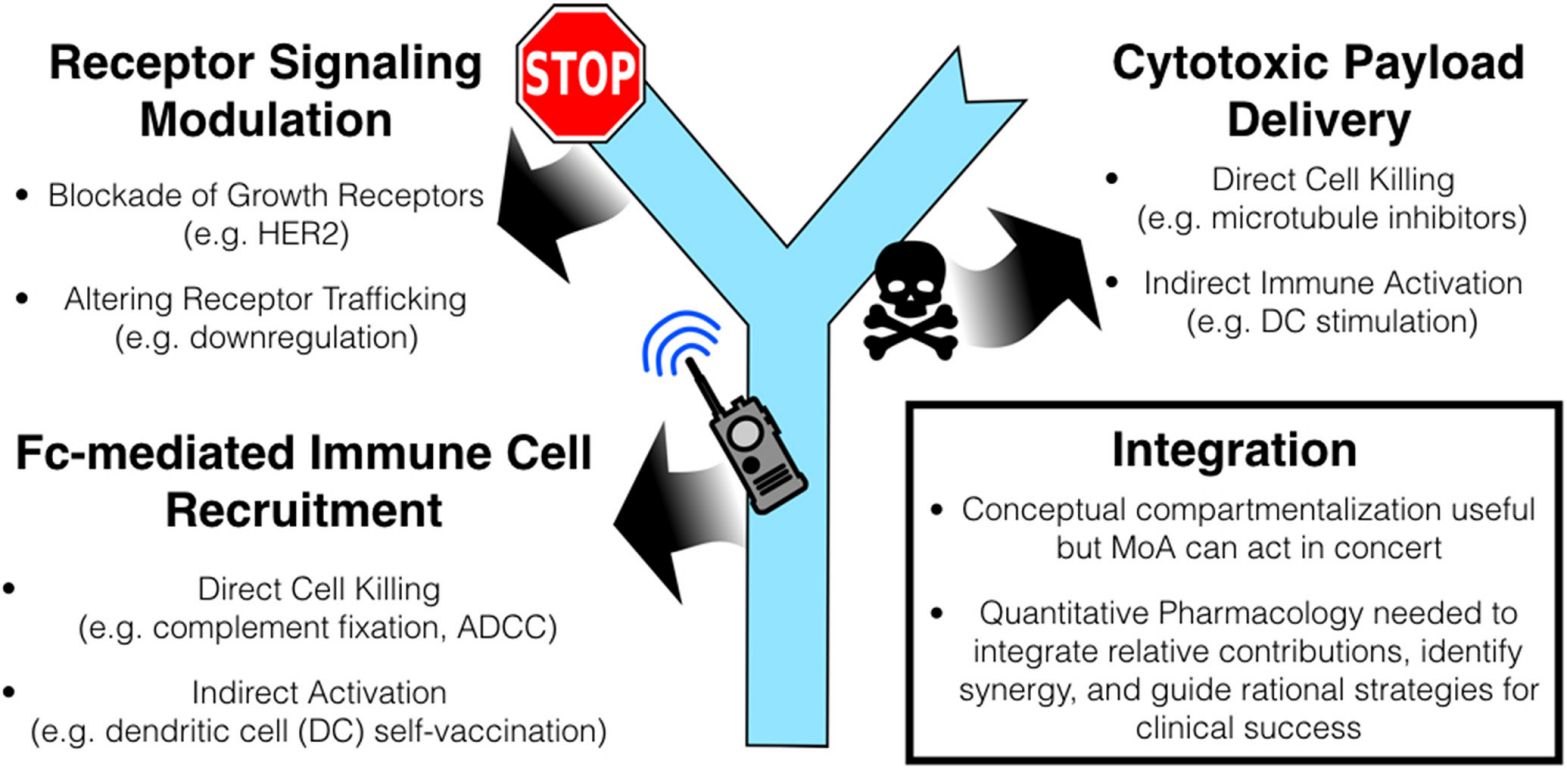
ADC Mechanisms of Action Integrating the contributions is necessary to identify the key attributes needed for clinical success.

There is a major push in drug development to efficiently generate clinically effective antibody-drug conjugates (ADCs), fueled by two recent FDA approvals. ADCs consist of a large monoclonal antibody (∼150 kDa) conjugated to potent, cytotoxic small molecule payloads through a chemical linker. These ‘hybrid’ drugs couple the properties of small molecule therapeutics with macromolecular biologics and function through multiple mechanisms of action (MoA). These include receptor-signaling modulation, cytotoxic payload delivery, and Fc-domain mediated functions such as antibody dependent cellular cytotoxicity (ADCC) and antigen presentation through dendritic cells (Figure 1). The various components driving each of these mechanisms, including target and payload selection, antibody properties (isotype, affinity, alternative scaffolds), linker, and dosing (Drug-Antibody Ratio/DAR, schedule), can dramatically shape the development of new agents. However, the relative contribution of each MoA to overall efficacy is generally unknown, particularly in the clinic. This leads to differing perspectives: some view ADCs as ‘targeted small molecules’ driven by the efficacy of the payload, whereas others view them as ‘armed antibodies’ leveraging antibody MoA. While this may first appear to be a semantic argument, quantifying the contribution from each distinct MoA to overall efficacy for this drug class is an essential step towards rationally guiding their clinical development.

The only FDA approved ADC for solid tumors, ado-trastuzumab emtansine (Kadcyla or T-DM1), is a prime example where each MoA is involved, but their relative contribution is unknown. Trastuzumab, the antibody backbone of T-DM1, blocks HER2 signaling within target cells, and presumably this contributes to the efficacy of T-DM1. On the contrary, T-DM1 is efficacious in relapsed patients previously treated with trastuzumab [1], indicating receptor signaling is not the only MoA influencing clinical efficacy. The Fc-mediated functions of trastuzumab contribute to efficacy, meaning immune cell recruitment and activation is another mechanism for T-DM1 efficacy, since it maintains Fc-effector functions. However, this does not necessarily identify Fc-effector functions as the critical MoA since the payload itself can contribute to immune cell activation [2], as seen with small molecule chemotherapeutics. Combined, these observations suggest all three MoA contribute to efficacy, but it is unknown if one MoA acts as a primary driver of clinical response or whether a combination/sum of effects is required.

Quantitative pharmacology can help resolve the contribution of each of these mechanisms and determine rational strategies to focus the development of next-generation ADCs. One of the most important parameters is clinical expression. IHC is the most common method for testing clinical expression, but each labeling protocol is different, so there is an urgent need to include internal controls to estimate absolute expression (targets per cell). Although IHC does not differentiate ADC-accessible from ADC-inaccessible target, it can help elucidate the widely varying expression levels in the clinic, ranging from less than a thousand to more than a million targets per cell. Antigen expression and ADC internalization rate determine the payloads delivered per cell, which along with the intrinsic payload potency establishes the overall potency of the ADC. T-DM1 is most effective in patients with 3+ staining [3], corresponding to ∼1 million HER2 receptors per cell. This suggests that concentrated cellular delivery of a potent payload is necessary for efficacy. If payload delivery versus potency is the critical factor in clinical efficacy, then next-generation compositional modifications, such as increased payloads per antibody, more potent payloads, and alternative scaffolds could drive more clinical success, particularly in patients with lower expression. However, clinical efficacy with high expression could also be indicative of an Fc-mediated response. Fc-domain density on the target cell surface helps determine ADCC activating signal strength, and high expression could elicit an immune response over repressive signals (e.g. IHC 3+ cell lines overcoming the repressive signal from glycans [5]). This MoA would support the use of combination therapies with immunomodulatory drugs such as checkpoint inhibitors to ‘tip the balance’ in favor of an immune response [6].

Carefully designed experiments using quantitative techniques in immunocompetent animal models will help dissect the relative importance of each MoA for a given target and design synergistic combination therapies. Strategies to maximize payload-driven tumor cytotoxicity include matching the cellular delivery to payload potency [7] and utilizing alternative scaffolds to increase tumor tissue penetration. If Fc-effector functions are a requisite for clinical activity (as determined by evaluations with Fc-mutants to isolate effector functions), then selecting high expressing targets/patients and/or pairing with immunomodulatory drugs [8] may be critical for clinical efficacy. Some strategies can improve multiple MoA. Recently, we demonstrated that increasing the antibody dose against highly expressed targets (at a constant payload dose) can improve payload delivery while increasing Fc-domain density on the target cell surface [4], potentially increasing Fc-mediated functions.

Dissecting the distinct contributions of ADC MoA, though challenging, can guide the design of clinically successful therapeutics. ADCs function through several MoA, and quantitative approaches can guide strategies such as “armed antibodies” where signal-mediated mechanisms dominate, “targeted small molecules” where cytotoxic payload-mediated mechanisms dominate, or a synergistic combination if all MoA are involved. Quantitative pharmacology can isolate these MoA, measure their relative contributions and trade-offs, and identify the primary driver(s) of clinical efficacy. For now, a vital first step towards rational design of ADCs is determining absolute targets/cell in the clinic. By measuring single-cell delivery, quantifying trafficking, and isolating mechanisms in immunocompetent animal models, robust ADC design principles can be developed to help focus ADC development and maximize their clinical efficacy.
